# Behind Closed Doors: The Priorities of the Alcohol Industry as Communicated in a Trade Magazine

**DOI:** 10.3389/fpubh.2018.00217

**Published:** 2018-07-31

**Authors:** Simone Pettigrew, Claire Hafekost, Michelle Jongenelis, Hannah Pierce, Tanya Chikritzhs, Julia Stafford

**Affiliations:** ^1^School of Psychology, Curtin University, Perth, WA, Australia; ^2^Telethon Kids Institute, The University of Western Australia, Perth, WA, Australia; ^3^McCusker Centre for Action on Alcohol and Youth, Curtin University, Perth, WA, Australia; ^4^National Drug Research Institute, Curtin University, Perth, WA, Australia

**Keywords:** alcohol industry, marketing strategies, promotion, policy implications, qualitative

## Abstract

**Background:** Efforts to reduce alcohol-related harm face strong resistance from the alcohol industry. It is important to monitor industry actions over time to assist in developing appropriate responses to this resistance. Monitoring can enable public health to identify industry positions on alcohol policy issues, stay abreast of current and emerging marketing tactics, and inform the development of possible counter-actions. One form of monitoring is the examination of industry trade publications where the industry converses with itself. The aim of this study was to assess industry strategic approaches as communicated in articles published in a leading Australian alcohol trade magazine to provide insights for policy makers and advocacy groups.

**Methods:** Thematic analysis of 362 articles published in a trade magazine over a one-year period.

**Results:** Three primary themes were evident in the articles: (1) the legitimization of alcohol as an important social and economic product, (2) the portrayal of the industry as trustworthy and benign, and (3) the strategic embedding of alcohol in various facets of everyday life.

**Conclusions:** There was a general failure to acknowledge the substantial burden of disease caused by alcohol products, and instead much effort was expended on legitimizing the product and the companies responsible for its production, distribution, and promotion. The level of denial exhibited shows that additional regulation of the industry and its tactics will need to proceed without industry acceptance. Clear resistance to increasing consumer protections also points to the futility of inviting industry members to the policy table.

## Introduction

Alcohol represents a single product category that is responsible for extensive harm globally. The World Health Organization (1, 2) lists alcohol consumption as one of the three primary causes of poor health and recommends that countries should attempt to achieve a minimum of a 10% decrease in harmful alcohol use by 2025. In Australia, the context of the present study, burden of disease figures show alcohol to be second only to tobacco in terms of fatal and non-fatal burden as measured by disability-adjusted life years (DALYs) (3). A recent national survey found that 18% of Australians aged 18+ years drink at levels associated with long-term harm (an average of more than 2 standard drinks per day) and 27% at levels associated short-time harm (more than 4 standard drinks on a single occasion at least once per month) (3). The cost to the Australian community of excessive alcohol consumption is estimated to be around $36 billion per year (4), which is far in excess of government revenues from alcohol taxes (estimated at $6.36 billion) (5). It is also substantially larger than the industry's estimated $12 billion in revenues (6). It is estimated that Australians consume an average of 9.7 liters of pure alcohol per year per capita (7). The huge prevalence and costs of alcohol-related harms is resulting in intensifying calls for increased regulation to limit how alcohol is supplied, priced, and promoted (8, 9).

Although the evidence base relating the adverse effects of excessive alcohol consumption is large (2, 10), and even moderate levels of intake have been associated with cancer risk (11), alcohol is promoted as a harmless lifestyle product (12, 13). Due to the nature and extent of the alcohol industry's marketing activities and the harmful nature of the product being promoted, comparisons are increasingly being made between the strategies used by alcohol and tobacco companies to attract and retain customers and to avoid or delay regulatory intervention [e.g., (14–16)]. Consistent with highly effective approaches in tobacco control, there is a need to monitor the alcohol industry's activities over time to determine appropriate courses of action (17). Such monitoring can assist public health to identify industry positions on alcohol policy issues, stay abreast of current and emerging marketing tactics, and inform the development of possible counter-actions.

Various research approaches to monitoring the marketing activities of the alcohol industry have been used to date. These include comparing the placement and content of alcohol promotion against mandatory and voluntary advertising regulations (18, 19), assessing the total quantity of alcohol advertising on television (13), determining the extent to which children are exposed to alcohol promotion (20), analyzing the intent and effectiveness of industry-developed “responsible drinking” messaging (21–23), evaluating the effectiveness of self-regulatory complaint review systems (24), and examining industry submissions to government policy consultations (25–28).

As the alcohol industry is unlikely to be transparent in disclosing their strategic intentions in public domains, alternative methods of accessing information need to be identified and deployed (29). One such method of obtaining information on the alcohol industry's current and future activities is a form of documentary analysis that involves reviewing the content of their trade magazines. Documentary analysis offers a means of providing data that can triangulate other forms of information generated on a specific topic (30). It has been applied in research relating to a broad range industries that have implications for public welfare. Prominent examples include the tobacco, food, beverage, gambling, and alcohol industries (25, 31–35). In the context of the present study, a documentary analysis approach was a means of obtaining alternative insights to those captured when observing the more public face the industry adopts when communicating directly with consumers and policy makers. When conversing with other in-group members, it is possible that different topics are raised and priorities expressed, potentially providing a clearer indication of marketing objectives and strategic directions. The aim of the present study was thus to thematically analyse the content of an Australian alcohol industry trade magazine to provide deeper insights into the ways the alcohol industry is approaching the marketplace.

## Method

The sample for the study comprised the editorial and advertorial content of all 11 issues of the Australian alcohol trade magazine, *National Liquor News*, for the calendar year 2015. The magazine calls itself “Australia's Leading Liquor Industry Magazine,” and at the time of data collection had a circulation of just over 12,000. As indicated by the nature of the articles and advertisements featured in the magazine, the publication has broad coverage of the alcohol industry, including producers, retailers, and promoters.

The editorial and advertorial content was collated for analysis by converting the articles into Word documents and then importing them into NVivo 11 (QSR International) for coding and analysis. Articles that were not included in the analysis featured industry-related content that was not relevant to the analysis (e.g., product advertisements and notifications of mergers and acquisitions, staff movements, and upcoming conferences). In total, 362 articles were included in the analysis.

An emergent coding hierarchy was used to assign article content to specific topics. This involved reading through numerous articles, developing an initial coding framework, and then progressively updating the framework as new topics became apparent. Coding was conducted at the line unit level, and NVivo's text function was used to retrospectively code previous content for topics that became apparent during the coding process. This grounded approach to data analysis (36) ensured that all article content was assigned to relevant codes. NVivo's matrix search function was subsequently used to generate frequencies of topic mentions and to assess prevalence of topics by different beverage types. The emergent nature of coding required the use of a single coder who engaged in peer debriefing with another author to refine the coding framework.

Once the coding was complete, a thematic approach was adopted to concisely describe the key issues represented by the identified topics. This involved forming cross-cutting themes that each encapsulated several of the individual topics in the form of conceptual clusters. The emergent themes were discussed and agreed among the author team.

## Results

Table 1 provides a summary of the content of the 362 coded magazine articles. The total number of articles featuring each topic is shown, along with a break-down by beverage type. Wine was the beverage type with the strongest presence in the articles, followed by beer. The topics that were most frequently mentioned were taste, quality, and pricing. Three primary cross-cutting themes were identified in the data that have potential implications for public health responses to the actions of the alcohol industry: (1) the legitimization of alcohol as an important social and economic product, (2) the portrayal of the industry as trustworthy and benign, and (3) the strategic embedding of alcohol in various facets of everyday life. Anonymised extracts from articles are provided to support the thematic analysis.

**Table 1:** Summary of magazine article content by alcohol type (*n* = 362 articles).

**Topic**	**Beverage type**									
	**Beer** ***n*** = **128**		**Cider** ***n*** = **47**		**Spirits** ***n*** = **112**		**Wine** ***n*** = **175**		**All**^∧^***n*** =**362**	
	***n***	%^*^	***n***	%^*^	***n***	%^*^	***n***	%^*^	***n***	%^*^
Awards	15	12	9	19	12	11	40	23	61	17
Craft/artisanal	93	73	22	47	31	28	32	18	97	27
Events	29	23	13	28	20	18	55	31	85	23
Family	5	4	3	6	16	14	51	29	69	19
Food pairing	21	16	3	6	16	14	38	22	59	16
Health and well-being	18	14	5	11	11	10	12	7	48	13
Novelty/Innovation	31	24	14	30	39	35	43	25	89	25
Pricing	42	33	13	28	32	29	59	59	104	29
Production processes	7	5	3	6	9	8	29	17	42	12
Quality	49	38	19	40	41	37	111	63	117	32
Regulation	11	9	2	4	9	8	14	8	38	10
Socializing	10	8	4	9	7	6	7	4	24	7
Sport	9	7	4	9	7	6	7	4	28	8
Taste	45	35	19	40	45	40	76	43	130	36

*∧Individual articles often referenced multiple topics and beverage types, which precluded significance testing*.

* *Percentages refer to the number of articles referring to a topic as proportion of the total number of articles mentioning a particular beverage type or all beverage types combined*.

### Theme 1: legitimizing alcohol as an important product

This theme relates to contributors' efforts to emphasize the role of alcohol products as a legitimate and important aspect of modern life. It incorporates the following codes from Table 1: quality, taste, pricing, production processes, novelty/innovation, craft/artisanal, and awards.

The analyzed articles provided deep insight into the extent to which the industry takes itself very seriously as a supplier of quality products that are of great importance to individual consumers and society in general. There were repeated descriptions of their product offerings enhancing people's lives in multiple ways:

*[Company name] will be taking a much more proactive role in the policy debate and will be actively promoting the benefits and contribution that the packaged liquor sector makes to the fabric of Australian society in social, employment, and economic terms, while providing consumers with a diverse choice of product range and outlets*.

*We are always experimenting and looking to inspire and delight people with creative and extraordinary ideas*.

Rather than viewing the industry as purveyors of largely mass-produced products that have the potential to cause substantial harm to users, the articles typically focused on specific product attributes and methods of production to highlight alcohol products as desirable, valuable, and sometimes artistic contributions to domestic and international marketplaces. Legitimacy was sought from a range of sources, including industry awards, sales data, and even academic research:

*A research project by the University of Adelaide has released some early results which show the positive feeling overseas wine trade and consumers have about Australian wine. When trade and consumer focus groups in the United States, United Kingdom, China, Korea, Indonesia, Vietnam and India were asked what they think about Australians and Australian wine they responded with descriptions of ‘authentic’, ‘exciting’, ‘sincere’, ‘strong’ and ‘reliable’…Our wines speak to the authenticity and uniqueness of our vineyards and the skills and passion of our grape-growers and winemakers, and it is very pleasing to learn that these messages can resonate internationally*.

However, despite the extensive discussions about product quality and consumers' quality of life, the articles were also clear that financial returns were the main driver. For example, economic developments in the marketplace were noted to be a primary source of innovation in the sector as industry members attempted to retain or grow their profits by developing attractive new product variations.

*The liquor industry continues to experience declining sales, as it has since the 1970s, but premiumisation offers relief by giving brands the opportunity to bolster sales revenue despite lower sales volume. Premiumisation is often thrown around, and with it yet to make the Macquarie dictionary, it's hard not to wonder if it's just meaningless jargon. Premium beer may have previously been judged on its ABV [alcohol by volume] or price point, but the recent proliferation of craft brands and explosion of various styles and prices means it's no longer a matter of strength or cost. Instead, mainstream brands are being pushed to follow the example set by more artisan craft brewers*.

This tension between presenting products as artistic contributions vs. profit-generating commodities was also evident in the manner in which alcohol consumers were described. Part of the justification for considering alcohol as a legitimate and important product category involved depicting customers as discerning individuals who expect and demand high quality alcohol products:

*Customers are increasingly interested in understanding the footprint of the product. Knowing the origin of the fruit which goes into our cider is undeniably a driving factor in them choosing to drink our cider, and it now has a ‘cult’ following of savvy urban cider drinkers*.

*Consumers want wines that tell a story and that are distinctive in their regional style and that are made by real people*.

This argument was often contradicted in other articles that focused on the need to educate consumers so they would come to demand the products the industry wants to sell:

*Consumers' palates become more sophisticated. However, they still need direction as to what styles and brands to buy, and gold medals or third-party endorsements play a strong role in consumer choice*.

*People need to be taught how to use liqueur*.

*In-store demonstrations and tastings are an essential way to both engage and educate drinkers*.

Other articles were more explicit in articulating the need to change consumers' perceptions about alcohol products to increase sales volume and profitability. In such arguments, consumers were depicted as ignorant of the “truth” and requiring attitudinal adjustment to enable the industry to achieve its sales objectives:

*The idea behind the initiative is to challenge some of the incorrect perceptions that people have about beer. [Person name] explained that research shows 88 per cent of consumers have said that they don't know exactly what goes into beer. She added that 71 per cent thought beer was fattening, 69 per cent thought it contained preservatives and that 94 per cent overestimated the sugar content in beer. By challenging and overcoming these perceptions, the Beer the Beautiful Truth campaign hopes to get people to reappraise beer and in doing that it presents a strong opportunity for growth. [Person name] highlighted that increasing beer sales will help to drive growth across all other categories. The campaign will receive $6 million in advertising over the next six months but [person name] added that the initiative will actually see a three-year investment aim to educate, inform and change perceptions of beer among Australian adults…The emotive reason is that we are in the beer business, we love beer and it's not very well understood and it gets a bad rap. So we wanted to educate people so they can reappraise beer. The rational part is that beer is at its lowest level of consumption per capita since figures have been recorded and that's a bit of an economic challenge for us, so if we can get people to reappraise beer and consume beer more frequently and enjoy beer more frequently it is going to be good for business*.

### Theme 2: we're the good guys

This theme relates to article content representing efforts to disparage any negative comment on the industry's activities, to quell arguments for increased regulation, and to paint themselves in a responsible light. It encapsulates the following codes from Table 1: regulation, health and well-being, and family.

There were numerous extended articles addressing the topic of regulation. These often featured a strong emotive style, with the authors expressing anger and frustration at any proposed regulatory intervention and exhibiting disbelief that such intervention could be warranted. Criticisms were levied at those advocating for additional protections for the community, and survey results and aggregated sales data were offered up as evidence that any arguments for additional regulation are fundamentally flawed:

*The problem for the alcohol industry is that governments do not measure their citizens' enjoyment and pleasure - governments only measure things that cost them money, such as ambulance callouts, hospital admissions, and police wages. So as far as a government is concerned, the ledger is always against us and more regulation would lower the government's costs*.

*The latest Alcohol Poll from the Foundation for Alcohol Research and Education (FARE) has been dismissed as “fear mongering” and “distorting public opinion”…Over recent years we have seen record levels of government funded behavioural research being used to perpetuate more fear and therefore more funding cycles in an environment where alcohol consumption is declining. Whilst the all-powerful and well-funded public health lobbyists would have Australians believe that drinking is at crisis levels, the opposite is true*.

*The legitimacy of the alcohol beverage industry is under significant threat as the social policy debate on the role of alcohol in Australian society is escalating in intensity. The dialogue is being dominated and misinformed by the more extreme elements of the public health anti-alcohol advocacy lobby*.

*ABS [Australian Bureau of Statistics] data shows Australians are drinking to success, not to excess*.

When blame wasn't being leveled at the government or health advocates, minority groups of drinkers were described as being at the root of the industry's reputational problems:

*Liquor store retailers and other industry members recognise the harms inflicted by a minority of irresponsible consumers*.

*There is not an Australian alive who would disagree that ending family violence and the very harmful abuse of alcohol by some Indigenous Australians would save many lives and improve our nation overall…(Two) reports called for an ending of alcohol sports sponsorships. How alcohol brands sponsoring sports teams worsens - or even mildly alters - the damaging and complex relationship between alcohol and those indigenous Australians who abuse alcohol is beyond understanding. The reports certainly didn't spell out the causal mechanism how that occurs*.

There were calls for the industry to unite in the face of this opposition:

*The liquor industry is a fantastic one to be part of, whether it be manufacturing, wholesaling or retailing through on or off- premise. There is a part to play for all of us in turning around the negative news bashing of our beloved industry and make sure we are seen in a more positive light*.

*We have so many intelligent, passionate and driven people in our industry if we band together we can take on anything that is thrown at us*.

Another approach was to focus on the ways the alcohol industry is supposedly working to assist Australians achieve higher levels of health and well-being. Certain forms of alcohol were pitched as being practical solutions for health- and safety-conscious drinkers.

*What's not to like about being able to enjoy a glass of wine with lunch and still be able to drive or not worry about dead calories? With winemakers putting considerable effort into creating wines with low ABV and calorie counts but high impact flavour profile and palates, it's a category definitely worth paying attention to*.

The attempts to link alcohol with a healthy diet included consulting a dietitian who focused only on the energy content of alcohol rather than other health-compromising attributes:

We speak to dietician [person name] about lower alcohol options. What are your thoughts on including alcohol as part of a healthy diet? “Alcohol can feature as part of a healthy diet - at the end of the day the word we all need to focus on more often is moderation. Many drinks contain minimal or no nutritional benefit but do still contain a relatively high number of calories or kilojoules.”

A final strategy to humanize the industry was to draw attention to the family connections of producers. This focus on a family orientation was often combined with the mention of other forms of social bonding:

*[Company name] is a fourth generation family wine company and continues the tradition of ‘good food, good wine and good times with family and friends’*.

*We are tangible and have real vines and real people with a great story to tell. We tell our story through the wine we make. We are honest, hardworking, down to earth people supporting local communities and families in the Great Southern region of Western Australia*.

### Theme 3: embedding alcohol in everyday life

This theme relates to efforts to create associations between alcohol consumption and a wide range of everyday activities to increase sales. It incorporates the following codes from Table 1: events, food, socializing, and sport.

A common approach in the articles was to emphasize the potential to capitalize on various contexts and occasions as catalysts for alcohol consumption. Numerous social events were listed as being prime opportunities to sell more alcohol, including barbecues, pubs, festivals, concerts, Christmas, New Year, Easter, Father's Day, St Patrick's Day, and in-store tastings. In some articles, entire months or seasons were considered to be drinking occasions.

Leveraging the key gifting periods of Father's Day, Christmas, and Easter…

*[Company name]'s strategy here is to continue its association with the Australian and international music culture and the promotion will see more than $2 million in music-related prizes given away*.

*This summer will see significant investment into both the off-trade and key venues as we continue to drive excitement and increase our association with the summer drinking occasion*.

Occasions involving food were often singled out as being especially fitting for the consumption of alcohol. Both casual eating occasions and more formal “celebrity chef” style events were described as being opportunities to ensure consumers understand the potential to include alcohol in any food-related experiences.

*Late last year we launched small batches of a seasonal series of ciders to pair with popular street food. The Street Food series concept was to allow people to grab a few bottles of cider, their favourite take-out and have an enjoyable casual dining experience with friends*.

*[Person name] recommends capitalising on the phenomenal growth in reality cooking shows and the related increase of awareness around food and beverage matching. Anything the retailer can do to stimulate these associations in-store will generate sales*.

Encouraging the pairing of alcohol with food was even positioned as a form of responsible marketing that enhances community well-being. Coincidentally, this approach also provides the opportunity to expand consumers' perceptions of the types of alcohol that are appropriate for serving with food:

*From our perspective, we must maintain our approach of responsible marketing to encourage and reflect community expectations for health and wellbeing, and that alcohol consumed responsibly has an important place in our modern society. Of course with the strong focus these days on food through cooking programs, restaurant reviews and celebrity chefs, the idea of wine and food as perfect partners is something to be promoted, and just as important is the idea that beers and spirits can be ideal bedfellows with food as well*.

Relatedly, linking brands to charities and causes was sometimes nominated as representing an “exciting” opportunity to enhance social welfare by partnering with respected groups to effectively communicate the message that alcohol and certain events are natural companions:

*[Company name] will sponsor the Prostate Cancer Foundation of Australia's (PCFA) 2015 Big Aussie Barbie campaign, which encourages Australians to turn their barbecues into fundraising events*.

Sporting events constituted a specific class of occasions that were clearly identified as an important source of alcohol promotion opportunities. Numerous different types of alcohol products were mentioned as being already involved in sponsorship arrangements that provide access to both existing and new consumer segments:

*The V8 Supercar sponsorship has helped drive our brand, with significant opportunity for consumers to try our products for the first time. We look forward to growing this relationship further for another five years with V8 Supercars*.

*[Company name] really wants to capitalize on our AFL [Australian Football League] and NRL [National Rugby League] property this year and we'll have some big news coming on the footy front. We'll be announcing some great promos that will have the attention of drinkers across the big sports months in autumn and winter*.

*There have also been some outstanding activations in Australia: Australian Open: [company name] accelerated its association with tennis at the most prestigious tennis event in Australia by announcing our partnership with world number one tennis player and three-time consecutive Australian Open winner, [person name]*.

## Discussion

The analyzed magazine editorial and advertorial content focused on positioning the alcohol industry and its products as legitimate, benign, and important elements of Australian life. This positioning is consistent with socio-cultural analyses of the role of alcohol in Australia that highlight the extent to which both every day and celebratory rituals include alcohol components that are considered essential to the experience (37–39). Numerous magazine articles referenced sharing alcohol with family and friends, especially at events involving the consumption of food and the enjoyment of music. Similarly, criticisms of alcohol advertising have emphasized the tendency for ads to depict alcohol as central to social interactions and the celebration of cultural events (12, 13, 40). There thus appears to be a situation of mutual reinforcement whereby the alcohol industry plays to existing social and cultural norms that favor the regular and frequent consumption of alcohol, potentially further solidifying their existence.

The positioning of alcohol consumption as normalized among large proportions of the population was found in the articles to be further justified through the argument that the majority of drinkers are moderate alcohol consumers and that minority groups or societal misfits who consume at very high levels are at fault for any negative public perceptions of the alcohol industry. This approach is likely to be an attempt by the industry to deflect attention away from the majority of “normal” drinkers (and sales). In fact, research demonstrates that the mean of alcohol use and the rate of heavy use in a population are closely related (41, 42). To reduce harms at population level, it is therefore necessary to reduce mean consumption for the whole population, not just among selected groups.

Overall, the results illustrate a general failure by the industry to appreciate and/or acknowledge the substantial burden of disease caused by their products. Also evident was resistance to additional forms of alcohol control that could serve to reduce the large costs of alcohol-related harm to individuals and the community. There is an apparent need for much greater acknowledgement of the substantial alcohol-related harms experienced in the community and the contribution of the industry to this state of affairs. The level of denial exhibited in the articles suggests that obtaining this acknowledgement will be a very difficult task and that additional regulation will need to proceed without industry acceptance. There are clear parallels with the arguments and tactics used by the tobacco industry as it came under increasing criticism as a source of substantial societal harm (15, 32). Specific examples include debating the accuracy and relevance of research evidence, citing endorsements from selected experts, increasingly shifting advertising expenditure to below-the-line promotion methods (e.g., sports sponsorship) as advertising restrictions are tightened, highlighting the contributions of the sector to the economy, and criticizing proposed policy changes.

### Implications for alcohol policy

Clear industry resistance to prioritizing public health over profits also points to the futility of involving industry members in the policy development process. A strong argument has been made that the vested interests of industries promoting unhealthy products prevent them from being appropriate participants in policy making (15, 43). By documenting the manner in which the industry communicates among its own members, the present study provides support for this exclusion argument. For example, dismissing ambulance callouts, hospital admissions, and police wages as purely economic costs of alcohol consumption and failing to appreciate the human tragedy behind these costs exemplifies the narrow perspective adopted by those who prioritize profits over people.

Recommendations for future alcohol policy are consistent and clear. There is strong support for minimum unit pricing, the introduction of a volumetric tax, mandatory advertising regulations, stricter trading hours limits, limiting outlet density, and alcohol warning labels (1, 44). The content of the trade magazine articles demonstrates that such interventions will be viewed by the industry as unreasonable incursions on their rights. But forewarned is forearmed, and the insights obtained from the articles provide some indication of effective approaches to address industry strategizing. First, while alcohol marketers attempt to better “educate” consumers to demand more of their products, the public health community needs to counter this with other forms of consumer education that draw drinkers' attention to alcohol-related harms. For example, research suggests that drinkers would be receptive to and influenced by warning labels that inform them that alcohol is a carcinogen (45–47). In addition, mass media campaigns along the lines of those used in tobacco control have the potential to ensure consumers are informed of the harms associated with alcohol consumption (48). This is important in the face of ubiquitous alcohol advertising that depicts alcohol as a harmless product (12, 13).

Second, some of the statements made in the magazines could constitute useful material to share with consumers to illustrate the nature of the industry's primary interests. Finally, the industry's efforts to embed alcohol in all possible social occasions highlight the need to provide alcohol-free contexts that de-normalize automatic associations between socializing and alcohol. This is especially critical in contexts where there are large numbers of young people who are being acculturated into adult society and learning about the role of alcohol via observation. Important starting points include policies that prevent adults from consuming alcohol at school events where children are present (49) and university policies that reduce students' exposure to alcohol on campus (50, 51). Other potential opportunities include policies relating to the service of alcohol at conferences and other work-related events (52).

In conclusion, this study provides insights into how the alcohol industry really thinks and acts. It is clear that profits dominate and social welfare is a distant, if not irrelevant, consideration. This should come as no surprise given that the primary responsibility of alcohol producers and distributors is to deliver profits to owners and shareholders. What is surprising is that the industry's views on regulation should receive the level of attention they currently enjoy. The persistence of voluntary alcohol advertising regulations is a case in point. The primary limitation of this study is the confinement to a single publication in just one country. Further monitoring of more varied alcohol trade journals in a broader range of national and cultural contexts could assist in enhancing and extending the interpretation provided here and provide representatives from the public health sector with a deeper understanding of how to anticipat e and address industry resistance to future efforts to reduce current high levels of alcohol-related harms.

## Ethics statement

The study was approved by the Curtin University Human Research Ethics Committee (approval number HRE2016-0513).

## Author contributions

JS and SP conceptualized the study. Data coding was undertaken by CH. SP took primary responsibility for analysing the data and preparing the manuscript. HP assisted with accessing the data. All authors contributed to the interpretation of the data and editing of the manuscript.

### Conflict of interest statement

The authors declare that the research was conducted in the absence of any commercial or financial relationships that could be construed as a potential conflict of interest.
